# Accurate Detection of Rifampicin-Resistant *Mycobacterium Tuberculosis* Strains

**DOI:** 10.3390/s16030376

**Published:** 2016-03-15

**Authors:** Keum-Soo Song, Satish Balasaheb Nimse, Hee Jin Kim, Jeongseong Yang, Taisun Kim

**Affiliations:** 1 Biometrix Technology, Inc. 202 BioVenture Plaza, Chuncheon 200-161, Korea; hanlimsk@empal.com; 2 Institute for Applied Chemistry and Department of Chemistry, Hallym University, Chuncheon 200-702, Korea; satish_nimse@hallym.ac.kr; 3 Korea Mycobacterium Resource Center (KMRC), The Korean Institute of Tuberculosis, Cheongju-si 28158, Korea; hatchingbird@yahoo.co.kr (H.J.K.); nonr@daum.net (J.Y.)

**Keywords:** tuberculosis, drug-resistant tuberculosis, diagnosis, accuracy, sensitivity, specificity

## Abstract

In 2013 alone, the death rate among the 9.0 million people infected with *Mycobacterium tuberculosis* (TB) worldwide was around 14%, which is unacceptably high. An empiric treatment of patients infected with TB or drug-resistant *Mycobacterium tuberculosis* (MDR-TB) strain can also result in the spread of MDR-TB. The diagnostic tools which are rapid, reliable, and have simple experimental protocols can significantly help in decreasing the prevalence rate of MDR-TB strain. We report the evaluation of the 9G technology based 9G DNAChips that allow accurate detection and discrimination of TB and MDR-TB-RIF. One hundred and thirteen known cultured samples were used to evaluate the ability of 9G DNAChip in the detection and discrimination of TB and MDR-TB-RIF strains. Hybridization of immobilized probes with the PCR products of TB and MDR-TB-RIF strains allow their detection and discrimination. The accuracy of 9G DNAChip was determined by comparing its results with sequencing analysis and drug susceptibility testing. Sequencing analysis showed 100% agreement with the results of 9G DNAChip. The 9G DNAChip showed very high sensitivity (95.4%) and specificity (100%).

## 1. Introduction

According to the Global tuberculosis report 2014, *Mycobacterium tuberculosis* (TB) is the world’s deadliest infectious diseases. In 2013 alone, the death rate among the 9.0 million people infected with TB worldwide was around 14%, which is unacceptably high. Furthermore, the Global tuberculosis report 2014 also pinpoints that the global proportion of new cases in 2013 with drug-resistant *Mycobacterium tuberculosis* (MDR-TB) was 3.5%, and this percentage is not changed in last few years [1]. The major factors behind the high prevalence rate of MDR-TB include poor access to laboratory diagnosis and effective treatment [2]. Therefore, the undiagnosed cases continue to spread the MDR-TB [3]. An empiric treatment of patients infected with TB or MDR-TB strain can also result in the spread of MDR-TB [4,5,6]. The approach of empiric treatment without the drug susceptibility testing (DST) in many developing countries is believed to aggravate the problem of MDR-TB in patients already infected with strains resistant to one or more drugs [7]. Therefore, a rapid and reliable diagnostic tool with simple experimental protocol can significantly help in decreasing the prevalence rate of MDR-TB strain.

A combination of rifampicin and isoniazid along with pyrazinamide and ethambutol is a standard treatment for TB infections. The rifampicin-resistant *M. tuberculosis* (MDR-TB-RIF) strains are known to have mutations in the *rpoB* gene [8]. There are at least 87 point mutations, short insertions, and deletions in the 81-bp core RRDR of *rpoB* codons 507 (c507) to 533 (c533) of MDR-TB-RIF strains [9]. The five codons (c511, c516, c522, c526, and c531) account for more than 90% of all mutations in the *rpoB* gene. Therefore, for monitoring the drug resistance, it is crucial to detect and discriminate the mutations in these codons [10]. The knowledge of rifampicin resistance will allow the clinicians to move onto second-line susceptibility testing and start empiric MDR-TB therapy [11,12]. Therefore, a diagnostic tool which is highly sensitive is of utmost importance in the fight against global tuberculosis crisis [13].

There are several methods in practice including DST, sequencing analysis, and real-time PCR. Each of these methods has their advantages and disadvantages. The phenotypic DST is a commonly used method for the identification of TB and MDR-TB-RIF strains. One of the drawbacks of this commonly used method is that it takes several weeks to obtain the final results [14,15]. The genotypic DST methods based on the molecular diagnostics are rapid. Genotypic DST methods are advantageous over phenotypic DST because they are rapid and have fewer laboratory biosafety restrictions [14]. DNA sequencing is a powerful tool for the detection of single nucleotide polymorphism (SNP) in the genomic DNA. Despite several advances in the field of sequencing analysis, it is inadequate for the routine clinical diagnosis because of the requirement of several sequencing reactions per sample making it labor-intensive and costly [16,17].

There are several reports on the detection and discrimination of TB and MDR-TB-RIF based on the real-time PCR technique. However, the problem of nonlinear amplification in real-time PCR results in a very low SNP discrimination ratio [18]. The GeneXpert MTB/RIF assay (Xpert MTB/RIF (Cepheid Inc, Sunnyvale, CA, USA)) uses hemi-nested real-time PCR-based technology and allows rapid detection of MDR-TB in clinical specimens [19]. However, GeneXpert MTB/RIF assay does not permit the detection of a particular mutation. On the contrary, DNA microarrays allow high-throughput detection of SNPs. However, conventional microarray-based detections have common drawbacks such as the use of high temperature (~60 °C), lengthier incubation time (3–12 h), low signal to background ratio (SBR) (3:1), and need of a large instrument [20,21].

In this article, we propose the use of 9G DNAChips for the highly sensitive and highly specific detection and discrimination of TB and MDR-TB-RIF strains. Recently reported 9G technology allowed the development of the 9G DNAChip [22,23,24]. The 9G DNAChip was designed to detect and discriminate multiple SNPs in the five codons (c511, c516, c522, c526, and c531) of TB strains at 25 °C, in less than 40 min after PCR. The clinical applicability of the 9G DNAChip for the detection and discrimination of TB and MDR-TB-RIF strains was evaluated by using 113 known cultured samples. The results of the 9G DNAChip were compared to the results of DST and sequencing analysis.

## 2. Materials and Methods

### 2.1. Materials and Instruments

Oligonucleotides, PCR pre-mix, and DNA extraction kits were purchased from Bioneer, Daejeon, Korea. Glass slides (2.5 by 7.5 cm) were procured from Paul Marienfeld GmbH & Co. KG, Lauda-Königshofen, Germany. High-performance liquid chromatography (HPLC) grade solvents were obtained from SK Chemicals, Seoul, Korea. A Milli-Q purification system (Millipore) was used for ultrapure water (18 MΩ/cm). Qarray2 microarrayer (Genetix Technologies, Inc., New Milton, UK) was used for oligonucleotide spotting. A commercial incubator and a commercial centrifuge (1000 rpm) were used. ScanArrayLite (GSI Lumonics) and Quant Array software (Packard Bioscience) were used for fluorescence signal measurement and image analysis, respectively.

### 2.2. Clinical Samples

The 113 cultured samples were acquired from the Korean Institute of Tuberculosis, Cheongju-si, Korea. All samples were previously evaluated for phenotypic DST by Löwenstein Jensen agar slants containing rifampicin [25]. Out of 113 samples, 103 samples were found to be MDR-TB-RIF strains and ten samples were rifampicin susceptible (TB strains). The genomic DNAs from the cultured samples were isolated according to the reported method [26]. The isolated DNAs were dissolved in the 100 mL of TE buffer, and the solution was used for the PCR amplification.

### 2.3. Asymmetric PCR Amplification

The single primer set (Forward Primer; 5’-GTC GCC GCG ATC AAG GAG TTC-3’, Cy5-Reverse Primer: 5’-Cy5-TCA CGT GAC AGA CCG CCG GG-3’) is used for the asymmetric PCR to generate amplicons of approximately 130bp as reported earlier [27]. The obtained PCR product was then used in the hybridization step. For the negative control, the PCR was performed without a template DNA.

### 2.4. Preparation of 9G DNAChip and Hybridization

**Figure 1:**
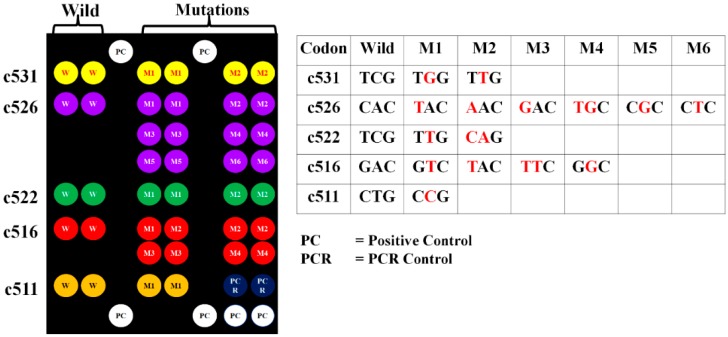
Respective positions of immobilized probes for codons c531, c526, c522, c516, and c511 (table on the right represents the corresponding wild and mutation sequence in these codons).

The probes required to obtain 9G DNAChips were selected by using generalized probe selection method [28]. A total of 22 probes were used to prepare 9G DNAChips based on the 9G technology [29]. In brief, the immobilization solutions were spotted on the AMCA (aminocalix[4]arene) slide using microarrayer to obtain 9G DNAChips (Figure 1). The immobilization solutions contained the probes corresponding to the five codons (c531, c526, c522, c516, and c511) of TB and MDR-TB-RIF strains along with the probes positive control (PC), and PCR control (PCR). For the detection of TB and MDR-TB-RIF strains, 20 μL of the Cy5 labeled PCR products were first mixed with a 50 μL of MDR-TB-RIF hybridization buffer solution. Then this mixture was loaded on the 9G DNAChips and incubated at 25 °C for 30 min in the commercial incubator. Then the 9G DNAChips were rinsed with MDR-TB-RIF washing buffer solutions A and B successively for 2 min each at 25 °C, and dried in a commercial centrifuge (1000 rpm). The images of fluorescence signals obtained by ScanArrayLite, were analyzed by Quant Array software.

The probes on the 9G DNAChip allow to detect and discriminate the wild and mutation sequences at codons c531, c526, c522, c516, and c511. As depicted in Figure 1, five wild probes corresponding to the five codons were used. However, two, six, two, four, and one probes were used for the detection of the mutations at the c531, c526, c522, c516, and c511, respectively. In the case of presence of TB DNA in a sample, only wild probes corresponding to the five codons show fluorescence signals. In the event of a sample containing genomic DNA of MDR-TB-RIF with a mutation in any one of these five codons, a signal is displayed by the corresponding mutation probe and no signal for the wild probe for that codon. In the case of the genomic DNA containing a mutation for which the 9G DNAChip does not have a probe, the absence of signal for the wild probe will indicate the presence of a mutation in that particular codon. Thus, the 9G DNAChip can detect TB and MDR-TB-RIF strains in the samples.

### 2.5. Sequencing Analysis

Sequencing analysis of the amplified *rpoB* gene fragment was used for the correct identification of TB and MDR-TB-RIF strains. The 439 base pair PCR product was obtained by using forward primer: 5′-TGGTCCGCTTGCACGAGGGTCAGA-3′ and reverse primer: 5′-GGC TAG CTT TGG GGA CTC CC-3′. The sequencing was performed as reported earlier [30].

## 3. Results

### 3.1. DST

Table 1 contains the outcome of DST. The 113 samples were chosen in such a way that 10 samples were TB, and 103 samples MDR-TB-RIF. However, particular mutations in the MDR-TB-RIF samples were unknown. Hence, these samples were considered as blind samples for the identification of mutations in the codons of the *rpoB* gene.

### 3.2. 9G DNAChip

**Figure 2:**
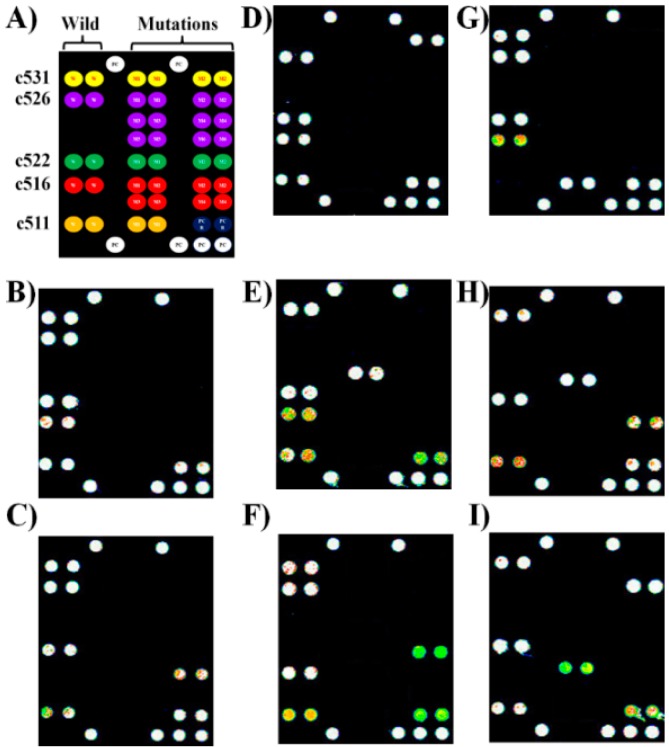
Multiplex detection of mutation in the multiple codons of the *rpoB* gene of TB in the clinical samples by rifampicin-resistant *M. tuberculosis* (MDR-TB-RIF) 9G DNAChip. (**A**) Scheme depicting the positions of immobilized probes; (**B**) Wild sample; (**C**) c516 (GAC→GTC); (**D**) c531(TCG→TTG); (**E**) c526 (CAC→CGC); (**F**) c522 (TCG→TTG); (**G**) c511(CTG→CCG); (**H**) c526 (CAC →CGC) and c511(CTG→CCG) and c526(CAC→CAA); (**I**) c516 (GAC→GTC) and c526 (CAC→CGC).

Figure 2 depicts the results of the independent experiments for the identification of mutation and discrimination of TB and the MDR-TB-RIF strains. A genomic DNA of wild TB strain in the clinical sample hybridizes with all five probes corresponding to five codons indicated by fluorescence signal. A genomic DNA of MDR-TB-RIF strain in the clinical sample hybridizes with a probe corresponding to the mutation codon indicated by a fluorescence signal (Figure 2B–H). All negative controls (without the genomic DNA) did not show a positive test for wild or mutant TB strain.

The outstanding specificity of the immobilize probes endowed by the generalized probe selection method, and controller DNA technology, ensured the SBR of more than 150:1 (Figure 2). The signal intensity in arbitrary units (A. U) for specific hybridization on the spots corresponding to each codon was in the range of the 50,000–60,000. Table 2 contains the results of 9G DNAChip for the detection of TB and MDR-TB-RIF strains in clinical samples. The 9G DNAChip detects the TB and MDR-TB-RIF strains by discriminating the mutations in the five codons of the *rpoB* gene. The detection of multiple SNPs in less than 40 min after PCR represents the high clinical applicability of the 9G DNAChip.

The results of 9G DNAChips and sequencing analysis for the detection and discrimination of mutations in the 103 MDR-TB-RIF samples were compared as depicted in Table 2. The samples that were found to contain the TB and MDR-TB-RIF strains in the sequencing analysis were also identified correctly by the 9G DNAChip. Out of the 113 clinical samples, 15 and 98 samples were found to be positive for TB and MDR-TB-RIF strains, respectively. Out of 98 samples 32 (31.1%), 24 (23.3%), 2 (1.9%), 29 (28.2%), and 4 (3.9%) samples were found to have mutations in c531, c526, c522, c516, and c511, respectively.

The details of the mutations found in the 103 rifampin-resistant samples by 9G DNAChip are presented in the Table 3. Out of 32 mutations samples corresponding to c531, one and 29 samples were found to have a single mutation as TCG→TGG and TTG, respectively. Remaining two samples were identified to contain a mutation at c531 because the wild probe corresponding to the c531 did not show any fluorescence signal. Out of 24 mutations samples corresponding to c526, seven, one, five, one, three, and four samples were found to have a single mutation as CAC→TAC, AAC, GAC, TGC, CGC, and CTC, respectively. Remaining three samples were identified to contain a mutation at c526 because the wild probe corresponding to the c526 did not show any fluorescence signal. Out of two mutation samples corresponding to c522, two samples were found to have a single mutation as TCG→TTG and TCG→CAG.

Out of 29 mutations samples corresponding to c516, 10, 15, and two samples were found to have a single mutation as GAC→GTC, TAC, and GGC, respectively. The remaining two samples were identified to contain a mutation at c516 because the wild probe corresponding to the c516 did not show any fluorescence signal. Out of four mutations samples corresponding to c511, three samples were found to have a single mutation as CTG→CCG. The other sample was confirmed to have a mutation at c511 because the wild probe corresponding to the c511 did not show any fluorescence signal. There were seven samples found to have mutations in more than one codon. Out of these seven samples five, and two samples had mutations in codons c516/c526, c526/c531, respectively. These results were in 100% agreement with sequencing analysis. There were five samples identified as false negative in the 9G DNAChip and sequencing analysis. The results of whole genome sequencing analysis indicated that there were mutations in the genes other than *rpoB* gene of the genomic DNA in these samples.

### 3.3. Sequencing Analysis

**Figure 3:**
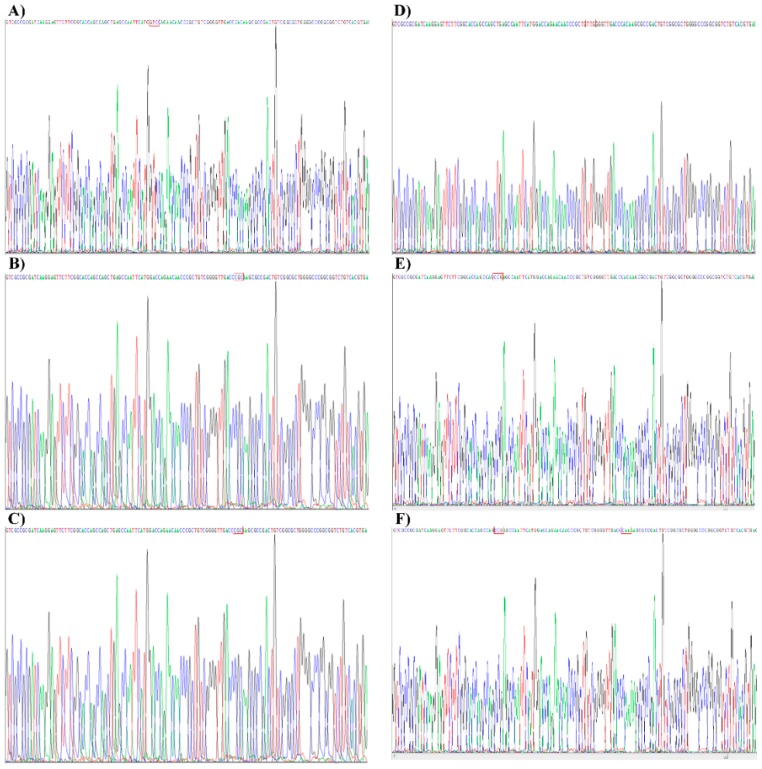
Examples of mutations identified by the sequencing analysis in the *rpoB* gene of TB in the clinical samples. (**A**) c516 (GAC→GTC); (**B**) c526 (CAC→CGC); (**C**) c531 (TCG→TTG); (**D**) c522 (TCG→TTG); (**E**) c511 (CTG→CCG); (**F**) c511 (CTG→CCG) and c526 (CAC→CAA).

The data of sequencing analysis were acquired in a format depicted in Figure 3. The outcome of the sequencing analysis of 113 clinical samples indicated the presence of TB and MDR-TB-RIF strains in 15 and 98 samples, respectively. Seven out of 98 samples were identified to have mutations in at least two codons of the *rpoB* gene, which was also detected by the 9G DNAChip. Out of seven samples, five and two samples were identified to contain mutations at c516/c526 and c526/c531, respectively. The results of sequencing analysis and 9G DNAChip were identical for the detection of mutations at codons c531, c526, c522, c516, and c511. However, sequencing analysis also showed the presence of the mutations in the other codons which were not covered by the 9G DNAChip.

There were 32 samples identified by the 9G DNAChip to contain a mutation in the c531. However, in sequencing analysis 24 (75%, TCG→TTC), one (3.1%, TCG→CAG), one (3.1%, TCG→GGG), and one (3.1%, TCG→TGG) samples were found to have a single mutation in c531. Five specimens (15.6%) containing multiple mutations, were found to have mutations in c531 and other codons which are not covered by 9G DNAChip. Similarly, out of 29 samples identified to contain mutation at c516 by 9G DNAChip, 10 (34.5%, GAC→GTC), 10 (34.5%, GAC→TAC), one (3.5%, GAC→GGC), and one (3.5%, GAC→CCC) samples were found to have a single mutation in c516. Seven samples (24.1%) were found to contain mutations at c516 and other codons.

Out of 24 samples identified to contain mutations at c526 by 9G DNAChip, six (CAC→TAC), five (CAC→GAC), two (CAC→CTC), one (CAC→CCC), one (CAC→TGC), and one (CAC→CGC) samples were found to have a single mutation in c526. Eight samples (15.6%) had mutations at c526 and other codons. Out of four samples identified to contain mutations at c511 by 9G DNAChip, one sample was found to have a mutation only at the c511 (CTG→CCG). However, other three samples had mutations at the c511 and other codons. There were only two samples identified to contain a single mutation at c522 (TCG→TTG, TCG→CAG) by 9G DNAChip and sequencing analysis. The whole genome sequencing of five samples falsely detected as negative both in the 9G DNAChip and sequencing analysis indicate the presence of the mutations in a gene other than the *rpoB* gene. Thus, 100% agreement is observed between the results of 9G DNAChip and sequencing analysis.

### 3.4. Statistical Analysis

To determine the, PPV, NPV, clinical sensitivity, and clinical specificity were calculated (Table 4) at 95% confidence intervals (CI). As depicted in Table 4, the sensitivity and specificity of the sequencing analysis and 9G DNAChip were found to be 95.4 (89.5–98.5) and 100 (69.2–100), respectively. Moreover, the PPV and NPV of the 9G DNAChip were found to be 100 (85.0–95.9) and 66.7 (38.4–88.18), respectively.

## 4. Discussion

The 9G DNAChip showed excellent performance concerning SBR and SNP discrimination ratios which were higher than 150:1. The 150 fold stronger signal for target hybridization than non-target hybridization allows 9G DNAChip to detect and discriminate the multiple mutations accurately. The 9G DNAChip showed higher sensitivity and specificity as compared to the reported methods [31,32]. It is important to notice that the sequencing analysis and 9G DNAChip showed 100% agreement for the detection of TB and MDR-TB-RIF strains. Apparently, the 9G DNAChip shows high accuracy in the detection of mutations.

Though the DST is a widely used technique, it is inappropriate for the rapid detection of TB and MDR-TB-RIF strains in the clinical samples. The DST is based on slowly growing mycobacterial cultures and takes several weeks. On the other hand, the 9G DNAChip takes only 40 min after PCR. Therefore, the 9G DNAChip is advantageous for diagnosis of TB and MDR-TB-RIF infections in the clinical samples because it is simple, rapid, and highly accurate.

The advances in the sequencing analysis have significantly accelerated the genomic research and discovery [33]. To date, sequencing analysis is the best method for the identification of the mutations in genomic DNA. However, it is not appropriate for the clinical applications because of the tedious experimental protocol, a requirement of highly trained professionals, and high cost per sample.

The Genotype MTBDR assay (Hain Lifescience GmbH, Nehren, Germany) and INNO-LiPA Rif. TB (Innogenetics, Ghent, Belgium) are reported for the detection of MDR-TB-RIF strains [34]. Unlike the 9G DNAChip and INNO-LiPA Rif. TB test, the Genotype MTBDR test can detect the presence of mutations in *rpoB* and katG genes. However, the 9G DNAChip has demonstrated its clinical applicability by showing 100% clinical sensitivity and specificity when compared with the sequencing analysis [35].

It is reported that the GeneXpert MTB/RIF test is also a sensitive method for the discrimination of TB and MDR-TB-RIF strains. The clinical sensitivity and specificity of the Xpert MTB/RIF test are 98.0 (98.0 to 99.0) and 99.0 (99.0 to 99.0), respectively [36]. However, the Xpert MTB/RIF test shows false-positive results, a common drawback associated with the real-time PCR methods [37]. The Xpert MTB/RIF test can detect the mutant codon but fails to identify the particular mutation in that codon. Recently reported MDR-TB-RIF 9G test [38] can also detect the mutant codon but does not give any information on the particular mutation in that codon. However, the 9G DNAChip not only detects the MDR-TB-RIF strains but also identifies the particular mutation the mutant codon. Therefore, the 9G DNAChip presented in this article and the MDR-TB-RIF 9G test are different from each other.

The high sensitivity and specificity for the detection of the TB and MDR-TB-RIF strains establish the accuracy of 9G DNAChip in the clinical diagnosis. It is considered that the following factors contribute to the proficiency of 9G DNAChip. (i) Implementation of the simple experimental protocol of hybridization, washing and drying at 25 °C all in 40 min; (ii) An SBR higher than 150:1 compared to 2.5 to 5 for the other microarrays or lateral flow assays; and (iii) 100% target specificity indicated by 100% concordance with sequencing analysis.

## 5. Conclusions

The SNP discrimination ratio and SBR higher than 150:1 endows the 9G DNAChip with exceptional sensitivity and specificity. The 9G DNAChip presented in this study is an excellent diagnostic tool as it can detect multiple SNPs and discriminates the TB and MDR-TB-RIF strains in the culture samples in less than 40 min after PCR. Furthermore, the 100% identical results in all cases with the sequencing analysis establishes the accuracy of the 9G DNAChip. The 9G DNAChip demonstrated very high sensitivity (95.4%) and specificity (100%). The PPV and NPV at 95% confidence interval were found to be 100% (85.0–95.9) and 66.7% (38.4–88.18), respectively. The 9G DNAChip demonstrates its clinical value as it can detect and discriminates TB and MDR-TB-RIF strains with 100% accuracy. The accuracy, efficiency and simplicity of the method for the detection and discrimination TB and MDR-TB-RIF strains in the culture samples assure the clinical applicability of 9G DNAChip.

## Figures and Tables

**Table 1:** Comparison of outcomes of the drug susceptibility testing (DST), sequencing analysis, and 9G DNAChip.

DST		Sequencing Analysis/9G DNAChip	
		Susceptible	Resistant
**Susceptible**	10	15	0
**Resistant**	103	0	98

**Table 2:** Outcomes of sequencing analysis and 9G DNAChip in 103 rifampin-resistant samples.

Mutation Codons	Number of Samples (%)	
	Sequencing Analysis	9G DNAChip
**c511**	4 (3.9%)	4 (3.9%)
**c516**	29 (28.2%)	29 (28.2%)
**c522**	2 (1.9%)	2 (1.9%)
**c526**	24 (23.3%)	24 (23.3%)
**c531**	32 (31.1%)	32 (31.1%)
**c516/c526**	5 (4.9%)	5 (4.9%)
**c531/c526**	2 (1.9%)	2 (1.9%)
Negative *	5 (4.9%)	5 (4.9%)

* Rifampicin resistant samples detected as TB strains in the sequencing analysis and 9G DNAChip.

**Table 3:** Specific mutations found in codons of *rpoB* gene by 9G DNAChip.

Codons	Samples	Original	Mutation (Number of Samples)
**c511**	4	CTG	CCG (3)
**c516**	29	GAC	GTC (10), TAC (15), GGC (2)
**c522**	2	TCG	TTG (1), CAG (1)
**c526**	24	CAC	TAC (7), AAC (1), GAC (5), TGC (1), CGC (3), CTC (4)
**c531**	32	TCG	TGG (1), TTG (29)
**c516/c526**	5	GAC/CAC	TAC/TAC (1), TAC/CGC (1), GTC/CAA (1), GTC/AAC (1), AAC/ AAC (1)
**c526/c531**	2	CAC/TCG	GGC/GCG (1), CTC/TTG (1)

**Table 4:** Performance of the 9G DNAChip.

Test	Sensitivity (95% CI)	Specificity (95% CI)	PPV (95% CI)	NPV (95% CI)
**Sequencing Analysis**	95.4 (89.5–98.5)	100 (69.2–100)	100 (85.0–95.9)	66.7 (38.4–88.18)
**9G DNACHIP**	95.4 (89.5–98.5)	100 (69.2–100)	100 (85.0–95.9)	66.7 (38.4–88.18)

## Abbreviations

The following abbreviations are used in this manuscript:

TB: *Mycobacterium tuberculosis*
MDR-TB: drug-resistant *Mycobacterium tuberculosis*
MDR-TB-RIF: rifampicin-resistant *Mycobacterium tuberculosis*
